# Association Between Self-Reported and Objective Activity Levels by Demographic Factors: Ecological Momentary Assessment Study in Children

**DOI:** 10.2196/mhealth.9592

**Published:** 2018-06-28

**Authors:** Jennifer Zink, Britni R Belcher, Eldin Dzubur, Wangjing Ke, Sydney O'Connor, Jimi Huh, Nanette Lopez, Jaclyn P Maher, Genevieve F Dunton

**Affiliations:** ^1^ Department of Preventive Medicine University of Southern California Los Angeles, CA United States; ^2^ Center for Outcomes Research Cedars Sinai Medical Center Los Angeles, CA United States; ^3^ Department of Kinesiology University of North Carolina at Greensboro Greensboro, NC United States

**Keywords:** sedentary behavior, physical activity, measurement, mobile devices, children

## Abstract

**Background:**

To address the limitations of the retrospective self-reports of activity, such as its susceptibility to recall bias, researchers have shifted toward collecting real-time activity data on mobile devices via ecological momentary assessment (EMA). Although EMA is becoming increasingly common, it is not known how EMA self-reports of physical activity and sedentary behaviors relate to the objective measures of activity or whether there are factors that may influence the strength of association between these two measures. Understanding the relationship between EMA and accelerometry can optimize future instrument selection in studies assessing activity and health outcomes.

**Objective:**

The aim of this study was to examine the associations between EMA-reported sports or exercise using the accelerometer-measured moderate-to-vigorous physical activity (MVPA) and EMA-reported TV, videos, or video games with the accelerometer-measured sedentary time (ST) in children during matched 2-h windows and test potential moderators.

**Methods:**

Children (N=192; mean age 9.6 years; 94/192, 49.0% male; 104/192, 54.2% Hispanic; and 73/192, 38.0% overweight or obese) wore an accelerometer and completed up to 7 EMA prompts per day for 8 days during nonschool time, reporting on past 2-h sports or exercise and TV, videos, or video games. Multilevel models were used to assess the relationship between the accelerometer-measured ST and EMA-reported TV, videos, or video games. Given the zero-inflated distribution of MVPA, 2-part models were used assess the relationship between the accelerometer-measured MVPA and EMA-reported sports or exercise.

**Results:**

EMA-reported TV, videos, or video games were associated with a greater accelerometer-measured ST (beta=7.3, 95% CI 5.5 to 9.0, *P*<.001). This relationship was stronger in boys (beta=9.9, 95% CI 7.2 to 12.6, *P*<.001) than that in girls (beta=4.9, 95% CI 2.6 to 7.2, *P*≤.001). EMA-reported sports or exercise was associated with a greater accelerometer-measured MVPA (zero portion *P*<.001; positive portion *P*<.001). This relationship was stronger on weekends, in older children, and in non-Hispanic children (zero portion all *P* values<.001; positive portion all *P* values<.001).

**Conclusions:**

EMA reports highly relate to accelerometer measures. However, the differences in the strength of association depending on various demographic characteristics suggest that future research should use both EMA and accelerometers to measure activity to collect complementary activity data.

## Introduction

Low physical activity (PA) is associated with cardiometabolic risk factors such as adiposity, insulin resistance, and elevated diastolic blood pressure in children [1]. Separate from PA, sedentary behaviors, typically accumulated in the form of screen time, are also associated with health consequences in children. For example, sedentary time (ST) is associated with higher body mass index (BMI) [2] and other cardiometabolic risk indicators such as increased triglyceride and blood glucose levels [3]. Furthermore, the combination of low levels of PA and high levels of ST may be particularly detrimental for children’s health because of the potentially synergistic nature of their health consequences [4]. To reduce morbidity and mortality, existing research has focused on gaining a better understanding of these health behaviors in children. However, research in this area is only as effective as the tools utilized to measure these variables.

Prior to the development of objective measures of activity, retrospective self-report measures, which ask participants to recall the intensity, duration, and frequency of activities over one or more days, were often utilized; however, these measures can be subject to recall errors and biases, especially in children [5,6]. Recalling PA and time spent engaged in sedentary behaviors is a demanding cognitive task for children, whose activities tend to be intermittent and vary in nature [5]. The field of behavioral health research shifted toward the use of device-based measures of activity, such as accelerometers, due to investigators becoming increasingly cognizant of the limitations of retrospective self-reports for activity data.

Accelerometers can be used to objectively quantify the frequency and duration of PA and ST in children [7] by detecting accelerations in movement [8]. These small hip- or wrist-worn devices are capable of measuring movement on 3 axes, vertical, anteroposterior, and lateral planes [8]. Thus, accelerometers are able to determine the frequency, duration, and intensity of movement in various directions and also do not require the cognitive demands of recalling activity behaviors, making them useful for measuring activity in children in a free-living environment [8]. However, there are some limitations to using accelerometers to assess PA and ST. Depending on their placement, accelerometers do not accurately capture upper body movements [9]. Furthermore, distinguishing wear time from ST can be a challenge when utilizing accelerometers [9]. Accelerometers also cannot provide insight with regard to the type of sedentary behaviors that are being performed by the participant. Evidence suggests that different types of sedentary behaviors, such as screen time and reading, have different relationships with physical and mental health indicators, such as BMI [10] and symptoms of emotional disorders in youth [11,12]. Thus, understanding the types of PA or sedentary behaviors undertaken is essential to investigate health outcomes. Therefore, accelerometers alone are limited in their ability to provide fully comprehensive information with regard to activity data.

To address the limitations associated with retrospective self-reports and to provide complementary activity data to accelerometers that otherwise would not be captured (eg, differentiating reading from sedentary screen time), investigators can use real-time self-report methods such as ecological momentary assessment (EMA) to assess the levels and types of PA and sedentary behaviors [13]. Using mobile technology, EMA methods address the limitations of retrospective self-report (eg, recall error and biases) by prompting participants to answer survey questions about recent behaviors occurring across limited time windows ranging from a few minutes to a few hours [14].

Traditional retrospective self-report methods of PA and sedentary behavior are only weakly correlated with device-based approaches such as accelerometry owing to the challenges described above [15]. Whether EMA reports provide measures of PA and sedentary behavior that are associated with accelerometer measures in children has yet to be tested in depth. Furthermore, multiple studies have indicated that correspondence between self-reports and accelerometer measures can differ based on demographic characteristics [16,17]; thus, investigating the role of potential modifiers is essential to optimizing measurement selection. Therefore, the aim of this study was to provide a preliminary assessment of the construct validity of EMA measures of structured leisure time PA (ie, sports or exercise) by comparing these measures with an accelerometer-measured moderate-to-vigorous PA (MVPA) in children. Evidence across multiple studies suggests that leisure time PA often occurs in the form of MVPA in youth [18,19]. Additionally, this study aimed to assess the construct validity of EMA measures of sedentary screen behaviors (ie, TV, videos, or video games) by comparing these measures with the accelerometer-measured ST in children. A secondary aim was to investigate whether the associations between the levels of activity measured by EMA and an accelerometer differ by child age, sex, ethnicity, or weight status; and on weekends versus weekdays—given that children’s levels of PA and time spent in sedentary behaviors can differ according to these variables [20,21].

## Methods

### Participants

Data were collected from children participating in the longitudinal observational Mothers’ and Their Children’s Health (MATCH) study. Baseline data were used for this analysis. The goal of the MATCH study was to examine the effects of maternal stress on obesity risk in children living in Southern California. Participant recruitment occurred via flyers and in-person research staff visits at public elementary schools and community events. The inclusion criteria for mother–child dyads were (1) the child is in the 3^rd^-6^th^ grade (aged 8-12 years), (2) more than half of the child’s custody belongs to the mother, and (3) both mother and child are able to read English or Spanish. Dyads were excluded from the study if the mother or the child (1) was taking medications for thyroid function or psychological conditions, (2) had a health condition that limited PA, (3) was enrolled in a special education program, (4) was currently using oral or inhalant corticosteroids for asthma, (5) was pregnant, (6) the child was classified as underweight by a BMI percentile <5% adjusted for sex and age, or (7) the mother worked more than 2 weekday evenings (between 5-9 pm) per week or more than 8 h on any weekend day. The MATCH study protocol is described in further detail elsewhere [22].

### Data Collection

Mothers provided in-person parental consent, and children provided written assent. Mothers completed paper and pencil questionnaires on their child’s age, sex, and race or ethnicity during a 90-min data collection session. Additionally, anthropometric measures of the child participants were taken at this time. Specifically, height (centimeters) and weight (kilograms) were collected in duplicate and averaged. Age- and sex-adjusted BMI z-scores were then calculated using the Centers for Disease Control EpiInfo 2005, Version 3.2 resource [22].

The children downloaded a custom-made EMA app for Android mobile phones (Google Inc., Mountain View, CA) on their personal mobile phones. If they did not have their own mobile phone, they were provided with a Moto G mobile phone (Motorola Mobility, Chicago, IL) to use for the duration of the study. After doing so, each child received random EMA prompts after 5:00 pm on the day of the data collection session (day 1) across the next 6 complete days (days 2-7) and up until 5:00 pm on the last day when the phone was returned to the researchers (day 8). On weekends, EMA surveys were prompted up to 7 times per day (between 7:00 am and 8:00 pm). On weekdays, EMA surveys were prompted up to 3 times per day (between 3:00 and 8:00 pm). Participants were instructed to proceed with their normal daily routines during the assessment period. The participant’s mobile phone would chime and vibrate to prompt the child to stop his or her current activity and answer EMA survey, which took approximately 2 min to complete. Assessments did not occur during school holidays or summer. At each prompt, children were asked: “In the past 2 HOURS, which of the following have you done? (choose all that apply).” Response options included “sports or exercise” and “TV, videos, and/or video games.”

Children were also provided Actigraph accelerometers (Model GT3X, Actigraph Corp., Pensacola, FL) and instructed to wear the devices on their right hip for the same 8 consecutive days as EMA data collection. MVPA was defined based on age-specific Freedson cut points [23], whereas ST was defined as <100 activity counts per minute [24]. Nonwear was defined as 60 min of consecutive zero count epochs [25], and only valid accelerometer wear time was used for this analysis. All accelerometer measurements were time-stamped so that they could be linked to the same time windows as EMA prompts. The University of Southern California Institutional Review Board approved all the procedures.

### Statistical Analysis

Frequencies and mean values were calculated for participant demographic characteristics and for EMA and accelerometer variables stratified across participant demographic characteristics and weekends versus weekdays. The mean and standard deviation of the accelerometer-measured activity stratified by yes or no EMA reports of PA (sports or exercise) and sedentary behavior (TV, videos, or video games) were also calculated. EMA prompt compliance was calculated as the proportion of prompts answered out of the total prompts. Additionally, multilevel logistic regression models were utilized to investigate whether age, sex, ethnicity, BMI-z, or weekends versus weekdays were associated with EMA prompt compliance (scored as yes or no). Linear mixed models were used to assess whether age, sex, ethnicity, BMI-z, or weekends versus weekdays were associated with nonvalid accelerometer time.

The relationship between the minutes of the accelerometer-measured ST within the last 2 h and EMA reports of sedentary screen behaviors within the same 2-h time window was investigated via linear mixed models using PROC MIXED in SAS v9.4 (SAS Institute, Cary, NC). Mixed models were used to adjust for the clustering of EMA responses that were nested within each child [26]. The dichotomous EMA reports of sedentary behaviors (ie, TV, videos, or video games) in the past 2 h were the independent variable, and the total minutes of the accelerometer-measured ST in the past 2 h were the dependent variable. All ST models were adjusted for age, sex, ethnicity, BMI-z, and weekends versus weekdays. These covariates were also tested as moderators by multiplying the main effect terms together to create 2-way interaction terms between EMA reports of ST with age, sex, ethnicity, BMI-z, and weekends versus weekdays. They were then entered into the models separately to test the significance of the interaction. Post hoc analyses were conducted where the models were stratified by any significant interaction variables identified. All models were also controlled for between-subject (BS) effects; this was done by creating BS and within-subject (WS) versions of the predictors to indicate an individual’s mean variation from the grand mean (using grand-mean centering) and one’s variation from his or her own mean (using person-mean centering) at any given prompt [27].

Traditional linear mixed models that assume a normal distribution are not appropriate for MVPA data because they are typically skewed with an inflated number of zero values [28]. Therefore, we used a 2-part model, which utilizes a mixture of logistic regression for zero MVPA values and gamma regression for positive MVPA values [28-30]. The logistic regression portion (zero portion) of the model predicts whether the participant was not active (odds of no activity), whereas the gamma regression portion (positive portion) predicts the expected amount of MVPA on occasions when the participant was active [28]. Thus, there are two interpretations of the results when utilizing this modeling method—the likelihood of no activity (zero portion) and the expected amount of activity when the participant was active (positive portion).

The 2-part models assessed the association between EMA reports of sports or exercise within the last 2 h and accelerometer-measured minutes of MVPA during that same 2-h time window using the “gsem” command in Stata 14.2. These models were adjusted for age, sex, ethnicity, BMI-z, and weekends versus weekdays. The aforementioned covariates were also tested as moderators by multiplying the main effects terms together to create 2-way interaction terms between EMA reports of PA with age, sex, ethnicity, BMI-z, and weekends versus weekdays. They were then entered into the models one at a time to test the significance of the interaction. Post hoc analyses were conducted, in which the models were stratified by any significant interaction variables identified. All models were also controlled for BS effects by creating BS and WS versions of the predictors using the same method, as previously mentioned [27].

## Results

### Description of Data Availability and the Study Sample

**Figure 1:**
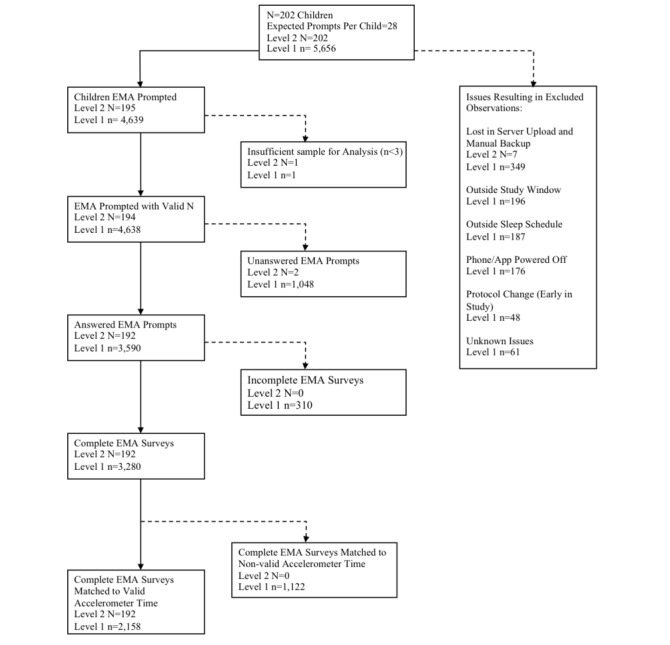
Solid Lines indicate available data, while dashed lines indicate data lost due to reasons indicated within each box. EMA: ecological momentary assessment.

Our sample consisted of 192 children with available EMA and accelerometer data of the 202 children, in total, enrolled in the MATCH study (Figure 1). As indicated by the flow diagram, exclusion may have occurred for a number of reasons, ranging from technical issues to unanswered EMA prompts. The mean (SD) age of the participants was 9.6 (0.9) years. Half (49.0%, 94/192) of the sample were boys, and 54.2% (104/192) were Hispanic. Of the participants, 38.0% (73/192) of the children were classified as overweight or obese based on their BMI-z. EMA prompt compliance was 75.7% (2158/2851), which is approximately the average level of compliance compared with EMA studies conducted on other samples of children [31]. A total of 157 participants (81.8%) completed 50% or more of possible EMA surveys, consistent with other studies on similar samples [32]. In total, 16 participants (8.3%) completed 100% of EMA surveys prompted during the assessment period of this study. Multilevel logistic regression analyses indicated that the likelihood of EMA prompt compliance was greater on weekends than on weekdays (odds ratio, OR 1.3, 95% CI 1.1 to 1.5). However, there were significantly more nonvalid accelerometer minutes on weekends than on weekdays (beta=16.9, *P*<.001). Additionally, as the child BMI-z score increased, there was a lower likelihood of EMA compliance (OR 0.8, 95% CI 0.7 to 0.9). No other demographic characteristics were significantly associated with EMA prompt compliance. No demographic characteristics were associated with nonvalid accelerometer time.

### Descriptive Statistics

The mean (SD) of the accelerometer-measured MVPA in the 2 h before EMA prompt was 10.4 (14.6) min. The mean (SD) of the accelerometer-measured ST in the 2 h before EMA prompt was 65.8 (21.2) min. Children reported sports or exercise in 37.4% (807/2158) of EMA prompts, and TV, videos, video games were reported in 47.3% (1021/2158) of prompts. Table 1 presents additional descriptive statistics on the accelerometer and EMA variables across participant demographic characteristics and on weekends versus weekdays, whereas Table 2 presents the mean (SD) accelerometer-measured activity stratified by EMA-reported sports or exercise and TV, videos, or video games.

**Table 1:** Descriptive statistics of accelerometer-measured activity and ecological momentary assessment (EMA)-reported activity during matched 2-h time windows stratified by demographic factors and weekends versus weekdays (Level 1 N=2158, Level 2 N=192).

Characteristic		Accelerometer-measured MVPA^a^ (minutes), mean (SD)	EMA-reported sports or exercise (yes), n (%)	Accelerometer-measured ST^b^ (minutes), mean (SD)	EMA-reported TV, videos, or video games (yes), n (%)
**Sex**					
	Boys	12.7 (18.7)	370 (36.2)	66.2 (22.2)	496 (48.5)
	Girls	8.3 (9.1)	441 (38.8)	65.8 (20.4)	531 (46.7)
**Age**					
	Above 9.6 years	8.28 (10.5)	451 (39.0)	68.3 (20.1)	543 (47.0)
	Below 9.6 years	12.9 (18.1)	360 (35.9)	63.2 (22.3)	484 (48.3)
**Ethnicity**					
	Hispanic	10.6 (16.7)	437 (38.8)	65.7 (21.8)	494 (43.9)
	Non-Hispanic	10.2 (12.0)	374 (36.2)	66.2 (20.7)	533 (51.7)
**BMI-z^c^**					
	Normal	11.6 (12.6)	538 (38.7)	65.5 (21.7)	691 (49.6)
	Overweight or Obese	8.3 (10.3)	273 (36.6)	66.7 (20.44)	336 (43.9)
**Weekend vs weekday**					
	Weekend	9.3 (14.6)	313 (31.7)	69.0 (21.9)	542 (54.9)
	Weekday	11.3 (14.7)	498 (42.6)	63.5 (20.5)	485 (41.5)

^a^MVPA: moderate-to-vigorous physical activity.

^b^ST: sedentary time.

^c^BMI-z: body mass index z-score.

**Table 2:** Descriptive statistics of the accelerometer-measured activity stratified by yes or no ecological momentary assessment (EMA) reports of sports or exercise and TV, videos, or video games during matched 2-h time windows (Level 1 N=2158, Level 2 N=192).

EMA-reported activity		Accelerometer-measured MVPA^a^ (minutes), mean (SD)	Accelerometer-measured ST^b^ (minutes), mean (SD)
**EMA-reported sports or exercise**			
	Yes	13.4 (15.9)	60.7 (20.6)
	No	7.6 (11.0)	69.3 (20.0)
**EMA-reported TV, videos, or video games**			
	Yes	8.0 (11.9)	70.1 (19.7)
	No	11.4 (14.4)	62.4 (20.7)

^a^MVPA: moderate-to-vigorous physical activity.

^b^ST: sedentary time.

### Associations Between EMA and Accelerometer Measures

Results from the linear mixed models investigating the relationship between EMA-reported sedentary behaviors and accelerometer-measured minutes of ST are shown in Table 3. Child EMA reports of engagement in the past 2-h TV, videos, or video games were associated with the greater minutes of the accelerometer-measured ST during the same time window (beta=7.3, 95% CI 5.5 to 9.0, *P*<.001). Additionally, the interaction between EMA-reported ST and sex was significant (beta=4.9, 95% CI 1.4 to 8.5, *P*=.01, Table 3); the strength of the association between EMA and accelerometer measures of ST was significantly different between boys and girls. Stratified post hoc analyses indicated that this association was twice as strong in boys (beta=9.9, 95% CI 7.2 to 12.6, *P*<.001) than that in girls (beta=4.9, 95% CI 2.6 to 7.2, *P*≤.001). No other significant moderators were found.

The results of the models investigating the relationship between EMA-reported sports or exercise and accelerometer-measured minutes of MVPA are shown in Table 4. The positive portion (the expected amount of activity measured by the accelerometer on occasions when the participant reported sports or exercise via EMA) and zero portion (likelihood of no activity measured by the accelerometer on occasions when the participant reported being active via EMA) of the 2-part model indicate a significant relationship between EMA and the accelerometer-measured activity in the total sample (zero portion estimate=−0.8, 95% CI −1.1 to −0.6, *P*<.001; positive portion estimate=0.6, 95% CI 0.5 to 0.7, *P*<.001). When the participant was active (according to the accelerometer) and reported sports or exercise via EMA in the last 2 h, this was associated with an 80.4% increase in the minutes of MVPA within that same time frame. Furthermore, when sports or exercise was reported via EMA, the odds of no MVPA measured by the accelerometer decreased by a factor of 56.8%.

The interaction term between EMA reports of sports or exercise and weekends versus weekdays was statistically significant, indicating a moderation of the association between EMA-reported sports or exercise and accelerometer-measured MVPA (zero portion estimate=−0.3, 95% CI −0.8 to 0.2, *P*=.24; positive portion estimate=0.2, 95% CI 0.05 to 0.4, *P*=.01). The relationship between the accelerometer-measured MVPA and EMA reports was stronger on weekends than on weekdays (Table 4). According to the positive portion of the model, if the participant had any accelerometer-measured MVPA, reporting sports or exercise via EMA was associated with a 103.4% increase in minutes of MVPA on weekends compared with only a 53.7% increase in minutes of MVPA on weekdays. Furthermore, when sports or exercise was reported via EMA, the odds of no accelerometer-measured MVPA decreased by 65.7% on weekends, whereas this was 30.2% on weekdays, according to the zero portion of the model.

In addition to weekends versus weekdays, age was found to be a significant moderator of the relationship between EMA-reported sports or exercise and accelerometer-measured MVPA (zero portion estimate=−0.1, 95% CI −0.4 to 0.2, *P*=.43; positive portion estimate=0.1, 95% CI 0.02 to 0.2, *P*=.02). This relationship was stronger in participants above the mean age of 9.6 years than in those below the mean age (Table 4). In children with an age above the mean age, if he or she had accelerometer-measured MVPA and reported sports or exercise, then there was a 101.4% expected increase in minutes of MVPA, whereas this expected increase was just 60% in children below the mean age, according to the positive portion of the model in each subsample. Furthermore, the odds of no accelerometer-measured MVPA decreased by a factor of 60.7% when exercise or sports were reported via EMA in children above the mean age compared with the reduced odds by a factor of 49.8% in children below the mean age, as indicated by the zero portions of each model.

**Table 3:** Coefficients with standard errors, 95% CI, and *P* values of mixed model with ecological momentary assessment (EMA) reports of sedentary screen behaviors as the predictor at level 1 on the accelerometer-measured sedentary time and mixed model with the significant interaction between EMA-reported sedentary screen behaviors and sex (Level 1 N=2158, Level 2 N=192).

EMA-reported activity		Model 1^a^			Model 2^a^		
		β (SE)	95% CI	*P*	β (SE)	95% CI	*P*
**Accelerometer-measured ST^b^with Level 1 predictor**							
	EMA-reported TV, videos, or video games	8.1 (0.9)	6.3 to 9.8	<.001	8.1 (0.9)	6.3 to 9.8	<.001
**Accelerometer-measured ST with Level 1 predictor adjusted for covariates at Level 2**							
	EMA-reported TV, videos, or video games	7.3 (0.9)	5.5 to 9.0	<.001	4.9 (1.2)	2.5 to 7.4	<.001
**Accelerometer-measured ST with cross-level interaction**							
	EMA-reported TV, videos, or video games x sex	N/A^c^	N/A	N/A	4.9 (1.8)	1.4 to 8.5	<.01

^a^The models are adjusted for sex, age, ethnicity, body mass index z-score, and weekends versus weekdays at level 2.

^b^ST: sedentary time.

^c^N/A: not applicable.

**Table 4:** Estimates with SE, 95% CI, and *P* values of the 2-part model with ecological momentary assessment (EMA) reports of leisure time physical activity predicting the accelerometer-measured MVPA in the total sample and stratified by the significant moderators of weekends versus weekdays, age, and ethnicity (Level 1 N=2158, Level 2 N=192).

EMA report of sports or exercise	Zero portion			Positive portion		
	Estimate (SE)^a^	95% CI	*P*	Estimate (SE)^a^	95% CI	*P*
Total sample (L1 N=2158)^b^	−0.8 (0.1)	−1.1 to −0.6	<.001	0.6 (0.1)	0.5 to 0.7	<.001
On weekends (L1 n=988)^b^	−1.1 (0.2)	−1.4 to −0.8	<.001	0.7 (0.1)	0.6 to 0.9	<.001
On weekdays (L1 n=1170)^b^	−0.4 (0.2)	−0.8 to 0.1	.10	0.4 (0.1)	0.3 to 0.5	<.001
Above 9.6 years old (L1 n=1156)^b^	−0.9 (0.2)	−1.2 to −0.6	<.001	0.7 (0.1)	0.6 to 0.8	<.001
Below 9.6 years old (L1 n=1002)^b^	−0.7 (0.2)	−1.1 to −0.3	.001	0.5 (0.1)	0.4 to 0.6	<.001
Non-Hispanic (L1 n=1032)^b^	−0.8 (0.2)	−1.2 to −0.4	<.001	0.7 (0.1)	0.6 to 0.9	<.001
Hispanic (L1 n=1126)^b^	−0.9 (0.2)	−1.2 to −0.5	<.001	0.5 (0.1)	0.4 to 0.6	<.001

^a^The models are adjusted for sex, age, ethnicity, body mass index z-score, and weekends versus weekdays.

^b^The abovementioned estimates have been exponentiated in the body of the paper for ease of interpretation.

Finally, ethnicity was also determined to be a significant moderator of the relationship between the accelerometer-measured MVPA and EMA-reported sports or exercise (zero portion estimate=−0.1, 95% CI −0.6 to 0.5, *P*=.86; positive portion estimate −0.2, 95% CI −0.4 to −0.1, *P*=.01). This relationship was stronger in non-Hispanic versus Hispanic children (Table 4). According to the positive portion of the model, if the participant had accelerometer-measured MVPA and reported sports or exercise, there was a 107.5% expected increase in minutes of MVPA in non-Hispanic children, whereas this increase in daily minutes of MVPA was only expected to be 61.6% in Hispanic children. The estimates from the zero portion of the model indicated that when the participant reported activity via EMA, the odds of no accelerometer-measured MVPA reduced by a factor of 56.0% in the non-Hispanic children. Similarly, these odds were reduced by a factor of 57.3% in the Hispanic participants. No other significant moderators were found between the accelerometer-measured MVPA and EMA reports of sports or exercise.

## Discussion

### Principal Findings

This is the first study comparing ST and MVPA measured concurrently by accelerometry and EMA-reported sedentary behaviors (ie, TV, videos, or video games) and PA (ie, sports or exercise) in children while also testing the moderators of the aforementioned relationships. Results indicated that EMA-reported sedentary behaviors were strongly positively associated with the accelerometer-measured minutes of ST during the same 2-h time frame. These findings indicate that EMA may be a promising method for capturing the specific forms of sedentary behavior through self-report with a very short-term recall window. Furthermore, EMA can provide contextual information such as where and with whom the behavior was performed [13]. The social and physical environments are important in understanding complex health behaviors, including sedentary behaviors [33]. For example, the built environment [34], peer relationships [35], and the day of the week [36] have all been shown to influence the levels of sedentary behavior in children. Other retrospective self-report tools for assessing sedentary behavior, such as the outdoor playtime recall questionnaire, are unable to provide such details surrounding sedentary behaviors and have demonstrated weak correlations with the accelerometer-measured ST [37,38]. Therefore, EMA may be more effective at capturing factors relevant to ST than other self-report measures previously utilized by investigators.

Although this evidence suggests that EMA is a helpful tool for gaining a better understanding of sedentary behavior in children, the results suggest that it may perform better in boys. The relationship between the accelerometer-measured ST and EMA reports of TV, videos, or video games was stronger in boys than in girls. These differences may emerge from the differences in leisure time sedentary behavior preferences in boys versus girls [39]. Studies indicate that boys spend more time playing computer games [40], whereas girls may prefer sedentary activities such as painting or drawing and playing musical instruments [39]. Therefore, EMA item capturing screen-based behaviors such as video games may have been a better indicator of the boys’ ST in our sample.

Results also showed a strong association between the accelerometer-measured MVPA and EMA reports of sports or exercise. When sports or exercise was reported in the past 2 h via EMA, significantly more minutes of MVPA were recorded by accelerometers during this time frame, and this was consistent with previous findings in adults [41]. A recent study comparing retrospective self-reports of PA and accelerometer-measured PA in youth found no relationship between the two [37]. Moreover, participants have been shown to overestimate the amounts of PA that they engaged in by an average of 596 minutes per week when utilizing retrospective questionnaires [17], highlighting the need for more effective self-report methods, particularly in children. The results of this study suggest that EMA self-reported PA highly relates to the accelerometer-measured PA, and children did not necessarily overestimate physical activity to the same degree when reporting via EMA. Secondary analyses of our data further support this notion; when there were zero minutes of the accelerometer-measured MVPA in the previous 2 h, participants in our sample only self-reported sports or exercise via EMA on 18% of occasions. Therefore, EMA has the potential to address the aforementioned weaknesses of other self-report PA measures by reducing the prevalence of over reports of PA.

However, when utilizing EMA as a PA data capture tool in children, investigators should be cognizant of time-variant and time-invariant variables that may influence the construct validity of EMA prompts. Specifically, the accelerometer-measured MVPA was more strongly related to EMA reports of sports or exercise on weekends than on weekdays. Multiple studies have demonstrated that the amount and types of PA that children participate in can differ between weekdays and weekends [36,42]. Children may be more likely to engage in nonrecreational types of PA during weekdays, such as active school transport (ie, walking), which may not be effectively captured by EMA item assessing leisure time PA in this study. Furthermore, a recent study conducted in more than 6200 children aged 9-11 years indicated that engaging in active school transport during the week was related to a greater accelerometer-measured MVPA during those days [43]. Therefore, activities such as active school transport during the week may contribute to the greater discrepancy observed between the accelerometer-measured MVPA and leisure time PA reported via EMA during weekdays.

Additionally, we found that the strength of the association between EMA self-reports of sports or exercise and accelerometer-measured MVPA differed between age groups. In those above the mean age of 9.6 years, the observed association between the two measures was stronger. In a study of more than 1000 active children aged 5-15 years, the investigators found that younger participants engaged in a more intermittent type of active play, whereas older children accumulated PA through walking and organized sport [44]. These differences in PA patterns may account for the age variations in the measurement associations that were observed within the current sample. Therefore, EMA item measuring sports or exercise, specifically, may be more successful at capturing the types of PA accumulated by older children, whereas the objective measures may be considered as the preferred method for capturing younger children’s physical activity behaviors, consistent with previous findings [45].

Finally, EMA reports of sports or exercise were more strongly related to the accelerometer-measured MVPA in non-Hispanic versus Hispanic children. In a nationally representative survey of children aged 9-13 years, it was determined that non-Hispanic children were significantly more likely to be involved in organized sports than their Hispanic counterparts [46]. To further support this notion, a large study of Hispanic children determined that first-generation Hispanic participants were less likely to report engaging in sports compared with their second- and third-generation peers [47]. Therefore, it may be that Hispanic children are accumulating PA through activities other than sports as a result of cultural preferences [47]. Thus, the current EMA items regarding leisure time sports or exercise may not be optimal for capturing PA behaviors in Hispanic participants.

This study highlights the strengths and weaknesses of EMA as a self-report tool for assessing leisure time PA and sedentary behavior data in children. Overall, EMA reports relate highly to the accelerometer-measured MVPA and ST. However, the moderators of this relationship reveal the limitations of EMA. EMA prompts asking about TV, videos, or video games might be a better indicator of ST in boys than in girls. Additionally, EMA prompts measuring sports or exercise appear to be a better indicator of PA on weekends than on weekdays. Finally, EMA self-reports of sports or exercise may be more effective for assessing PA in older (above 9.6 years old in our sample) and non-Hispanic children. To address the limitations of EMA, investigators may tailor EMA items to capture the types of PA and sedentary behaviors typically performed by individual participants in their samples. If tailoring EMA items is not feasible, the moderators of the relationship between EMA reports and objectively measured activity levels should be considered when analyzing and interpreting EMA data. As a general recommendation, it is also suggested that future investigators utilize both accelerometers and EMA simultaneously, depending on the scope of the investigation. It may be more useful to utilize EMA in studies assessing different types of behaviors (eg, reading vs computer use) that are being performed at any given moment, whereas accelerometers may be more useful in circumstances when investigators are interested in the overall frequency or duration of activity behaviors. Informed instrument selection will ultimately increase our understanding of PA and sedentary behaviors and how they relate to preventable health issues.

### Limitations

Although our study has several strengths, there are limitations to note. Depending on their placement, accelerometers cannot detect all bodily movements such as upper body activities, and their detection of activity is sensitive to chosen cut points. Furthermore, EMA responses may be subject to recall bias, though to a lesser extent, compared with the traditional retrospective self-report strategies [13]. Therefore, a study comparing two tools that have inherent limitations may ultimately be considered as a weakness. Additionally, we were not able to assess whether the amount of time elapsed between EMA prompt and the provision of an answer moderated EMA-accelerometer associations in this study. Furthermore, children were not EMA-prompted before 3 pm on weekdays; therefore, the results from this study may not generalize the behaviors and activities performed during school hours. In addition to this, the first prompt after 3 pm on weekdays asked participants about their behaviors “since you woke up this morning,” as opposed to asking about the previous 2-h behaviors. Therefore, the behaviors reported during this EMA prompt may not reflect the past 2-h activity levels measured via accelerometer. However, post hoc exploratory analyses removing the first prompts of weekdays (the prompts asking about behaviors since the participant woke up that morning) minimally altered our parameter estimates and results. This pattern suggests that these EMA items did not influence our findings.

Another limitation of this study is that contextual (eg, environmental or social) data were not assessed, and this may influence EMA-accelerometer associations; future studies should attempt to address this limitation. EMA prompt compliance was greater on weekends and in children with lower BMI-z. Thus, our findings may not be as generalizable to data captured on weekdays as well as data collected in heavier participants. Additionally, there was more nonvalid accelerometer time on weekends, which presents as an additional limitation for generalizability. Finally, our sample from Southern California metropolitan community, which contains more than 50% Hispanic participants, may differ from the general population of youth living in the United States and therefore may limit the generalizability of our results. Future studies should attempt to address these generalizability issues.

### Conclusions

Findings indicate that EMA reports of TV, videos, or video games were strongly related to the accelerometer-measured ST during the same 2-h time frame. However, this relationship was stronger in boys than in girls. Although EMA reports of sports or exercise were associated with the accelerometer-measured MVPA, time-variant (weekends vs weekdays) and -invariant (age and ethnicity) variables were found to be the moderators of this relationship. EMA reports of sports or exercise and accelerometer-measured MVPA were more strongly associated on weekends, in older children, and in non-Hispanic participants. These moderators can be addressed by tailoring EMA items designed to capture PA and sedentary behaviors based on specific participant demographics and the day of the week. Taken together, this study supports EMA as a useful self-report tool for capturing PA and sedentary behavior in children because it demonstrates a high correlation with objectively measured activity levels.

## Abbreviations

BMI: body mass index
EMA: ecological momentary assessment
MVPA: moderate-to-vigorous physical activity
PA: physical activity
ST: sedentary time
